# Association of Plasma Matrix Metalloproteinase and Tissue Inhibitors of Matrix Metalloproteinase Levels With Adverse Treatment Outcomes Among Patients With Pulmonary Tuberculosis

**DOI:** 10.1001/jamanetworkopen.2020.27754

**Published:** 2020-12-01

**Authors:** Nathella P. Kumar, Kadar Moideen, Arul Nancy, Vijay Viswanathan, Kannan Thiruvengadam, Shanmugam Sivakumar, Syed Hissar, Dina Nair, Vaithilingam V. Banurekha, Hardy Kornfeld, Subash Babu

**Affiliations:** 1 National Institutes of Health–National Institute for Research in Tuberculosis–International Center for Excellence in Research, Chennai, India; 2 National Institute for Research in Tuberculosis, Chennai, India; 3 Prof M. Viswanathan Diabetes Research Center, Chennai, India; 4 Laboratory of Parasitic Diseases, National Institute of Allergy and Infectious Diseases, National Institutes of Health, Bethesda, Maryland

## Abstract

**Importance:**

Identifying biomarkers of treatment response is an urgent need in the treatment of tuberculosis (TB). Matrix metalloproteinases (MMPs) and tissue inhibitors of matrix metalloproteinases (TIMPs) are potential diagnostic biomarkers in pulmonary TB (PTB).

**Objective:**

To assess whether baseline plasma levels of MMPs and TIMPs are also prognostic biomarkers for adverse treatment outcomes in patients with PTB.

**Design, Setting, and Participants:**

Two different cohorts (test and validation) of individuals with PTB were recruited from 2 different sets of primary care centers in Chennai, India, and were followed up for treatment outcomes. Participants were individuals with newly diagnosed TB that was sputum smear and culture positive and drug sensitive. A total of 68 cases and 133 controls were in the test cohort and 20 cases and 40 controls were in the validation cohort. A nested case-control study was performed by matching case patients to control participants in a 1:2 ratio for age, sex, and body mass index. Data for the test cohort was taken from a study performed from 2014 to 2019, and data for the validation cohort, from a study performed from 2008 to 2012. The data analysis was performed from November 2019 to May 2020.

**Interventions:**

Individuals with PTB were treated with antituberculosis chemotherapy for 6 months and followed up for 1 year after completion of treatment.

**Main Outcomes and Measures:**

Individuals with PTB with adverse outcomes (treatment failure, all-cause mortality, or recurrent TB) were defined as cases and those with favorable outcomes (recurrence-free cure) were defined as controls. Plasma levels of MMPs and TIMPs were measured before treatment as potential biomarkers.

**Results:**

In all, 68 cases and 133 matched controls were enrolled in the study (170 [85%] males and 31 [15%] females; median age, 45 years [range, 23-73 years]) in the test cohort and 20 cases with 40 matched controls (51 [85%] males and 9 [15%] females; median age, 45 years [range, 19-61 years]) in the validation cohort. Baseline plasma levels of 5 MMPs and 2 TIMPs in the test cohort and 5 MMPs and all 4 TIMPS in the validation cohort were significantly higher in cases vs controls. In the test cohort, the geometric means (GMs), cases vs controls, were as follows: for MMP-1, 3680 vs 2484 pg/mL (*P* = .008); for MMP-2, 6523 vs 4762 pg/mL (*P* < .001); for MMP-7, 3346 vs 2100 pg/mL (*P* < .001); for MMP-8, 1915 vs 1066 pg/mL (*P* < .001); for MMP-9, 2774 vs 2336 pg/mL (*P* = .009); for TIMP-1, 4491 vs 2910 pg/mL (*P* < .001); and for TIMP-2, 3082 vs 2115 pg/mL (*P* < .001). In the validation cohort, the GMs, cases vs controls were as follows: for MMP-1, 3680 vs 2484 pg/mL (*P* < .001); for MMP-2, 6523 vs 4762 pg/mL (*P* < .001); for MMP-7, 3346 vs 2100 pg/mL (*P* < .001); for MMP-9, 1915 vs 1066 pg/mL (*P* < .001); for MMP-13, 2774 vs 2336 pg/mL (*P* < .001); for TIMP-1, 4491 vs 2910 pg/mL (*P* = .003); for TIMP-2, 3082 vs 2115 pg/mL (*P* = .003); for TIMP-3, 2066 vs 1020 pg/mL (*P* < .001); and for TIMP-4, 2130 vs 694 pg/mL (*P* < .001). Plasma levels of MMPs and TIMPs were associated with increased risk of adverse outcomes according to both univariate and multivariable analysis in the test cohort (eg, univariate analysis: odds ratio [OR] for MMP-8, 2.04; 95% CI, 1.33-3.14; *P* = .001; multivariable analysis: OR for MMP-8, 2.16; 95% CI, 1.34-3.47; *P* = .001). Combined receiver operating characteristic analysis revealed significant area under the curve (AUC), with high sensitivity and specificity in both cohorts (eg, for a combination of MMP-2, MMP-7, and TIMP-1 in the test cohort: sensitivity, 84%; specificity, 83%; and AUC, 0.886; for a combination of MMP-2, MMP-7, TIMP-1, and TIMP-2 in the validation cohort: sensitivity, 85%; specificity, 95%; and AUC, 0.944).

**Conclusions and Relevance:**

Baseline plasma MMP and TIMP levels may be correlates of risk and prognostic biomarkers for treatment failure, relapse, and death in individuals with PTB and merit further evaluation as predictive biomarkers for stratification of patients to shortened or intensified treatment regimens.

## Key Points

**Question:**

Are baseline plasma levels of matrix metalloproteinases (MMPs) and tissue inhibitors of matrix metalloproteinases (TIMPs) prognostic biomarkers of adverse treatment outcomes in individuals with pulmonary tuberculosis?

**Findings:**

This case-control study of 68 case patients and 133 control patients in a test cohort and 20 cases and 40 controls in a validation cohort of newly diagnosed patients with culture-positive, drug-sensitive pulmonary tuberculosis found that baseline plasma levels of MMPs and TIMPs were associated with increased risk of adverse treatment outcomes and may serve as prognostic biomarkers of treatment outcomes.

**Meaning:**

Baseline plasma MMP and TIMP levels merit further evaluation as prognostic biomarkers for stratification of patients to type of tuberculosis treatment.

## Teaser

This case-control study examines the value of matrix metalloproteinases (MMPs) and tissue inhibitors of matrix metalloproteinases (TIMPs) as biomarkers of treatment outcomes among patients with pulmonary tuberculosis.

## Introduction

India accounts for 26% of all cases of tuberculosis (TB) worldwide and ranks as the No. 1 country for TB burden.^1^ The World Health Organization and the Indian government have set ambitious goals for the elimination of TB.^2,3^ However, to achieve this goal, new studies are needed, including studies to identify biomarkers of treatment response.^4^ A 6-month regimen of 4 drugs (4 drugs in the first 2 months and 2 drugs in the next 4 months) is the standard of care for drug-susceptible pulmonary TB (PTB) based on the need to achieve low recurrence rates, yet less than 20% of patients with TB are expected to be at increased risk for recurrence if treatment shortening to 4 months or less is implemented.^5^ Treatment shortening would greatly aid in the progress toward elimination of TB by promoting adherence, favoring better case management and disease control, minimizing the risk of acquired drug resistance, and lowering the operational burden on national programs.^6,7^ Thus, the need to identify biomarkers of cure that could allow stratification of patients at risk for adverse treatment outcomes is urgent.^4^

Matrix metalloproteinases (MMPs) are a family of proteolytic enzymes with various physiologic roles that have been postulated to play a key role in the pathogenesis of PTB.^8,9^ Matrix metalloproteinases have been implicated in the pathology of lung tissue damage and cavitation because they have the ability to degrade all fibrillary components of the extracellular matrix.^10,11^ They help spread *Mycobacterium tuberculosis* from the lung parenchyma to the airways and thereby facilitate transmission.^10,11^ Elevated MMP levels are associated with various markers of TB disease severity, including sputum smear status, radiographic evidence of disease extent, and cavitary disease.^12,13,14,15^ In addition, MMPs are thought to modulate inflammation.^16^ Tissue inhibitors of matrix metalloproteinases (TIMPs) are endogenous regulators of MMPs^17^ and have also been shown to be important biomarkers of TB disease severity.^18,19^ Both MMPs and TIMPs are also biomarkers of extrapulmonary TB.^20^

We hypothesized that baseline plasma levels of MMPs and TIMPs would be associated with adverse treatment outcomes among patients with PTB. To test this hypothesis, we examined the baseline levels of MMPs and TIMPs in 2 different nested case-control studies of favorable vs adverse treatment outcomes in a cohort of individuals with PTB in Chennai, India.

## Methods

The test cohort was recruited from a study approved by the Ethics Committees of the Prof. M. Viswanathan Diabetes Research Center and National Institute for Research in Tuberculosis (NIRT). The validation cohort was recruited from a study approved by the internal ethical committee of NIRT. Informed written consent was obtained from all participants. All the methods were performed in accordance with the relevant institutional ethical committee guidelines. This study was conducted in accordance with the amended Declaration of Helsinki.^21^ This report was prepared according to the Strengthening the Reporting of Observational Studies in Epidemiology (STROBE) reporting guideline.

### Study Population

The test cohort of 446 individuals comprised all participants enrolled in the Effect of Diabetes on Tuberculosis Severity study,^22^ a prospective cohort study (2014-2019) conducted in Chennai, India, who had a poor treatment outcome such as treatment failure, relapse, or death (n = 68). Ten percent of participants (n = 44) were lost to follow-up. The validation cohort included 82 individuals enrolled in the study on Immune Responses in Pulmonary Tuberculosis (2008-2012)^23^ conducted in Chennai, India; 20 of these individuals had a poor treatment outcome such as treatment failure, relapse, or death and 12% (n = 9) were lost to follow-up. The inclusion criteria were new positive findings on smear and culture, age between 18 and 75 years, and no previous treatment for TB. Exclusion criteria for both cohorts were previous treatment for TB, drug-resistant TB, HIV seropositivity, or current immunosuppressive drug therapy. Additional exclusion criteria for the validation cohort were diabetes mellitus, hypertension, alcoholism, smoking, and low or high body mass index (BMI) (calculated as weight in kilograms divided by height in meters squared). The diagnosis of PTB was established by positive sputum culture results on solid media with compatible findings on a chest radiograph. Anti-TB treatment was based on Directly Observed Treatment, Short course (DOTS) therapy.^24^ Participants were followed up monthly through the 6-month course of treatment and every 3 months thereafter until 1 year after treatment completion. We conducted a nested case-control study: case patients who had adverse treatment outcomes were matched in a 1:2 ratio to control participants, who were defined as having a recurrence-free cure until the end of study. Cure was defined as negative results of sputum cultures at months 5 and 6 of treatment without recurrent disease during follow-up. Adverse treatment outcomes included treatment failure defined as positive sputum culture results at month 5 or 6, all-cause mortality, or recurrent TB within 12 months after initial cure. Eighteen treatment failures, 16 deaths, and 34 recurrences occurred in the test cohort. Eight treatment failures and 12 recurrences occurred in the validation cohort. Case-control matching was carried out on the basis of age, sex, BMI, and diabetes status. All cases and controls were exactly matched for age and sex. Body mass index was matched within 1 index unit of BMI. Diabetes status was determined by a response of yes or no from each participant. Peripheral blood samples were collected in heparinized tubes. After centrifugation, plasma samples were collected and stored at −80 °C until further analysis could be done. Samples were collected at baseline (before treatment initiation).

### Metalloproteinase Assays

Circulating plasma levels were measured using a commercially available kit (Luminex Magpix Multiplex Assay system; Bio-Rad). The MMP and TIMP levels also were measured using commercially available kits (Luminex Human Magnetic Assay kit 8 Plex and Luminex Human Magnetic Assay kit 4 Plex; both R&D Systems). The lowest detection limits were as follows: MMP-1, 23.87 pg/mL; MMP-2, 91.7 pg/mL; MMP-3, 77.9 pg/mL; MMP-7, 78.4 pg/mL; MMP-8, 84.9 pg/mL; MMP-9, 118.3 pg/mL; MMP-12, 9.2 pg/mL; MMP-13, 211.3 pg/mL; TIMP-1, 14.6 pg/mL; TIMP-2, 45.9 pg/mL, TIMP-3, 133 pg/mL; and TIMP-4, 7.2 pg/mL.

### Statistical Analysis

The data analysis was performed from November 2019 to May 2020. Geometric means (GMs) were used for measurements of central tendency. Differences between case and control groups were analyzed using the Mann-Whitney test. Analyses were performed using Prism, version 8.0 (GraphPad). To define the plasma MMP and TIMP levels that correlate with adverse treatment outcomes, we performed univariate and multivariable conditional regression analyses, with the latter corrected for age, sex, BMI, diabetes status, smoking, alcoholism, presence of cavitation, smear and culture status, and socioeconomic status. Univariate and multivariable conditional logistic regression analyses were fitted with the variables as potential predictors, using TB treatment outcome as the main outcome variable. Before analysis was performed, the presence of multiple collinearities was considered. Data analysis was performed using STATA software, release 15.0 (StataCorp). Graphical representation was made using Prism software. All *P* values were 2 sided, with statistical significance evaluated at the.05 α levels. To assess whether we could use plasma levels of MMPs and TIMPs as predictive biomarkers of adverse treatment outcomes in patients with PTB, we performed a combined receiver operating characteristic (ROC) analysis of various MMP and TIMP levels in both the test and validation cohorts to evaluate their ability to discriminate cases and controls. We performed a combinatorial analysis of multiple immune biomarkers to define ideal marker combinations of the tested circulating plasma MMPs and TIMPs using the CombiROC method. We created all possible MMP and TIMP combinations for each poor treatment outcome group with respect to controls in both the test and validation cohorts. The combinations that delivered the highest sensitivity and specificity values were considered for selection of efficient immune biomarker signatures. Computation and selection of optimal biomarker combinations by integrative ROC were analyzed using a freely available web application, CombiROC, version 1.2.^25^

## Results

### Study Population

The details of the study population in the test and validation cohorts are provided in Table 1 and the eTable in the Supplement, respectively. The test cohort (68 cases and 133 matched controls) consisted of 170 (85%) men and 31 (15%) women; their median age was 45 years (range, 23-73 years). The validation cohort (20 cases and 40 matched controls) included 51 (85%) men and 9 (15%) women; their median age was 45 years (range, 19-61 years). There were no significant differences in age, sex, BMI, diabetes status, lipid profile, alcoholism, educational level, or socioeconomic status in the test cohort. There were no significant differences in age or sex in the validation cohort. There were no differences in smear or culture grades or in the presence of cavitation in the 2 cohorts.

**Table 1.** Demographic and Clinical Characteristics of the Test Cohort

Characteristic	No. (%)			*P* value
	All (N = 201)	Cases (n = 68 [34%])	Controls (n = 133 [66%])	
Age, median, y	45 (23-73)	45 (23-65)	45 (25-73)	.27
Sex				
Male	170 (85)	60 (88)	110 (82)	.21
Female	31 (15)	8 (12)	23 (18)	
BMI, median (range)	3.1 (12.7-30.1)	3.04 (12.8-25.1)	3.15 (12.7-30.1)	.15
DM status				
DM	116 (58)	42 (61)	74 (56)	.41
No DM	85 (42)	26 (39)	59 (44)	
CXR score, median (range)	38 (2-130)	38 (5-130)	37 (2-125)	.19
Cavity				
Yes	54 (26)	18 (26)	36 (26)	.94
No	118 (59)	40 (59)	78 (59)	
Unknown	29 (15)	10 (15)	19 (15)	
Smear grade^a^				
1+	126 (63)	36 (53)	90 (67)	.06
2+	67 (33)	27 (40)	40 (30)	
3+	8 (4)	5 (7)	3 (3)	
Culture grade^b^				
1+	86 (43)	25 (37)	61 (47)	.16
2+	36 (18)	10 (15)	26 (19)	
3+	79 (39)	33 (48)	46 (34)	
Dyslipidemia				
Yes	73 (36)	25 (36)	48 (36)	.93
No	128 (64)	43 (64)	85 (64)	
Smoking				
Yes				.03
Current	61 (30)	28 (41)	33 (25)	
Former	40 (20)	14 (21)	26 (20)	
No, never	100 (50)	26 (38)	74 (55)	
Alcoholism				
Yes				.47
Current	105 (52)	39 (58)	66 (50)	
Former	38 (19)	13 (19)	25 (18)	
No, never	58 (29)	16 (23)	42 (32)	
Education				
Educated	160 (80)	53 (78)	107 (80)	.68
Uneducated	41 (20)	15 (22)	26 (20)	
Occupation				
Unemployed	14 (7)	4 (6)	10 (7)	.56
Unskilled worker	102 (51)	40 (59)	62 (47)	
Skilled worker	53 (26)	16 (24)	37 (28)	
Business or professional	11 (6)	3 (4)	8 (6)	
Retired or housewife	21 (10)	5 (7)	16 (12)	

Abbreviations: BMI, body mass index (calculated as weight in kilograms divided by height in meters squared); CXR, chest radiograph; DM, diabetes mellitus.

^a^ Under 200× magnification, 1+ indicates 3 to 24 acid-fast bacilli (AFB) in 1 field; 2+, 25 to 250 AFB in 1 field; and 3+, more than 250 AFB in 1 field.

^b^ Under 200× magnification, 1+ indicates 10 to 100 colonies; 2+, more than 100 to 200 colonies; and 3+, more than 200 colonies.

### Association of MMP and TIMP Plasma Levels With Correlates of Risk in the Test Cohort

**Figure 1.**
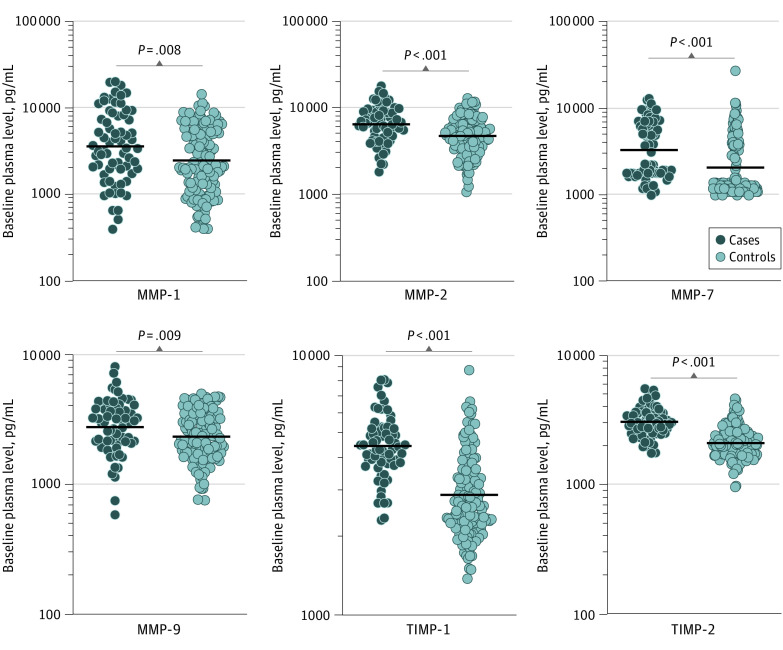
Elevated Baseline Plasma Levels of Matrix Metalloproteinases (MMPs) and Tissue Inhibitors of Matrix Metalloproteinases (TIMPs) in Cases in the Test Cohort The baseline plasma levels of MMPs and TIMPs were measured in cases (n = 68) and controls (n = 133). The data are represented as scatterplots, with each circle representing a single individual and the heavy horizontal line representing the median. *P* values were calculated using the Mann-Whitney test with the Holm correction for multiple comparisons.

To determine the baseline levels of plasma MMP and TIMP levels in cases and controls, we measured the expression of MMPs and TIMPs before treatment in the test cohort. As shown in Figure 1 and eFigure 1A in the Supplement, plasma levels of MMP-1 (GM of 3680 pg/mL in cases vs 2484 pg/mL in controls), MMP-2 (GM of 6523 pg/mL in cases vs 4762 pg/mL in controls), MMP-7 (GM of 3346 pg/mL in cases vs 2100 pg/mL in controls), MMP-8 (GM of 1915 pg/mL in cases vs 1066 pg/mL in controls), and MMP-9 (GM of 2774 pg/mL in cases vs 2336 pg/mL in controls) were significantly higher in cases compared with controls, whereas the plasma levels of MMP-3 (GM of 2139 pg/mL in cases vs 2865 pg/mL in controls) were significantly lower in cases. Similarly, the plasma levels of TIMP-1 (GM of 4491 pg/mL in cases vs 2910 pg/mL in controls) and TIMP-2 (GM of 3082 pg/mL in cases vs 2115 pg/mL in controls) were significantly higher in cases compared with controls. Univariate analysis showed that MMP-1 (odds ratio [OR], 1.36; 95% CI, 1.01-1.85; *P* = .04), MMP-2 (OR, 2.76; 95% CI, 1.40-5.48; *P* = .004), MMP-7 (OR, 1.75; 95% CI, 1.18-2.61; *P* = .006), MMP-8 (OR, 2.04; 95% CI, 1.33-3.14; *P* = .001), TIMP-1 (OR, 7.55; 95% CI, 2.85-19.9; *P* < .001), and TIMP-2 (OR, 12.50; 95% CI, 3.98-41.82; *P* < .001) were associated with increased risk of adverse treatment outcomes, whereas MMP-3 (OR, 0.48; 95% CI, 0.27-0.88; *P* = .02) was associated with a decreased risk of adverse outcomes. Multivariable analysis showed that MMP-1 (adjusted OR [aOR], 1.32; 95% CI, 0.95-1.84; *P* = .10), MMP-2 (aOR, 3.26; 95% CI, 1.51-7.03; *P* = .003), MMP-7 (aOR, 1.71; 95% CI, 1.09-2.68; *P* = .02), MMP-8 (aOR, 2.16; 95% CI, 1.34-3.47; *P* = .001), MMP-9 (aOR, 2.16; 95% CI, 0.99-4.67; *P* = .051), TIMP-1, (aOR, 8.23; 95% CI, 2.92-23.22; *P* < .001), and TIMP-2 (aOR, 14.31; 95% CI, 3.83-53.39; *P* < .001) were still associated with a significantly increased risk of adverse treatment outcomes, whereas MMP-3 (aOR, 0.45; 95% CI, 0.22-0.92; *P* = .03) was still associated with a significantly decreased risk of adverse outcomes (Table 2). Thus, baseline plasma levels of MMPs and TIMPs may be correlates of risk for adverse treatment outcomes in patients with PTB in the test cohort.

**Table 2.** Association of the Baseline MMP and TIMP Levels With Treatment Outcomes

Marker	Univariate model		Multivariable model	
	OR (95% CI)	*P* value	aOR (95% CI)^a^	*P* value
MMP-1	1.36 (1.01-1.85)	.04	1.32 (0.95-1.84)	.10
MMP-2	2.76 (1.40-5.48)	.004	3.26 (1.51-7.03)	.003
MMP-3	0.48 (0.27-0.88)	.02	0.45 (0.22-0.92)	.03
MMP-7	1.75 (1.18-2.61)	.006	1.71 (1.09-2.68)	.02
MMP-8	2.04 (1.33-3.14)	.001	2.16 (1.34-3.47)	.001
MMP-9	1.80 (0.89-3.64)	.10	2.16 (0.99-4.67)	.051
MMP-12	1.15 (0.30-4.41)	.84	1.29 (0.30-5.50)	.73
MMP-13	0.20 (0.02-2.39)	.20	0.18 (0.01-2.77)	.22
TIMP-1	7.55 (2.85-19.9)	<.001	8.23 (2.92-23.22)	<.001
TIMP-2	12.50 (3.98-41.82)	<.001	14.31 (3.83-53.39)	<.001
TIMP-3	0.94 (0.29-3.02)	.92	0.75 (0.23-2.47)	.64
TIMP-4	1.01 (0.62-1.63)	.98	1.00 (0.59-1.70)	>.99

Abbreviations: aOR, adjusted odds ratio; MMP, matrix metalloproteinase; OR, odds ratio; TIMP, tissue inhibitor of matrix metalloproteinases.

^a^ Multivariable conditional logistic regression models were used to study the association of biomarker with treatment outcomes (unfavorable) and are adjusted for age in years, sex, body mass index, diabetes status, smoking status, alcohol status, and smear grading.

### Association of MMP and TIMP Plasma Levels With Correlates of Risk in the Validation Cohort

**Figure 2.**
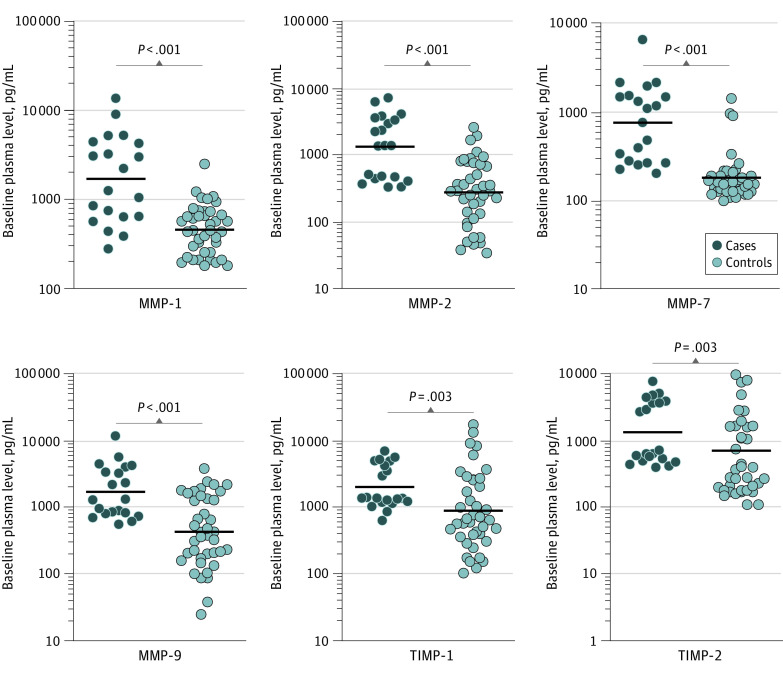
Elevated Baseline Plasma Levels of Matrix Metalloproteinases (MMPs) and Tissue Inhibitors of Matrix Metalloproteinases (TIMPs) in Cases in the Validation Cohort The baseline plasma levels of MMPs and TIMPs were measured in cases (n = 20) and controls (n = 40). The data are represented as scatterplots, with each circle representing a single individual and the heavy horizontal line representing the median. *P* values were calculated using the Mann-Whitney test with the Holm correction for multiple comparisons.

To assess the baseline levels of plasma MMP and TIMP in cases and controls, we measured the expression of MMPs and TIMPs before treatment in the validation cohort. As shown in Figure 2 and eFigure 1B in the Supplement, plasma levels of MMP-1 (GM of 3680 pg/mL in cases vs 2484 pg/mL in controls), MMP-2 (GM of 6523 pg/mL in cases vs 4762 pg/mL in controls), MMP-7 (GM of 3346 pg/mL in cases vs 2100 pg/mL in controls), MMP-9 (GM of 1915 pg/mL in cases vs 1066 pg/mL in controls), and MMP-13 (GM of 2774 pg/mL in cases vs 2336 pg/mL in controls) were significantly higher in cases compared with controls. Similarly, the plasma levels of TIMP-1 (GM of 4491 pg/mL in cases vs 2910 pg/mL in controls), TIMP-2 (GM of 3082 pg/mL in cases vs 2115 pg/mL in controls), TIMP-3 (GM of 2066 pg/mL in cases vs 1020 pg/mL in controls), and TIMP-4 (GM of 2130 pg/mL vs 694 pg/mL in controls) were also significantly higher in cases compared with controls. Thus, baseline plasma levels of MMPs and TIMPs were significantly elevated in patients with adverse treatment outcomes in the validation cohort.

### MMPs and TIMPs as Predictive Biomarkers of Adverse Treatment Outcomes in Patients With PTB

**Figure 3.**
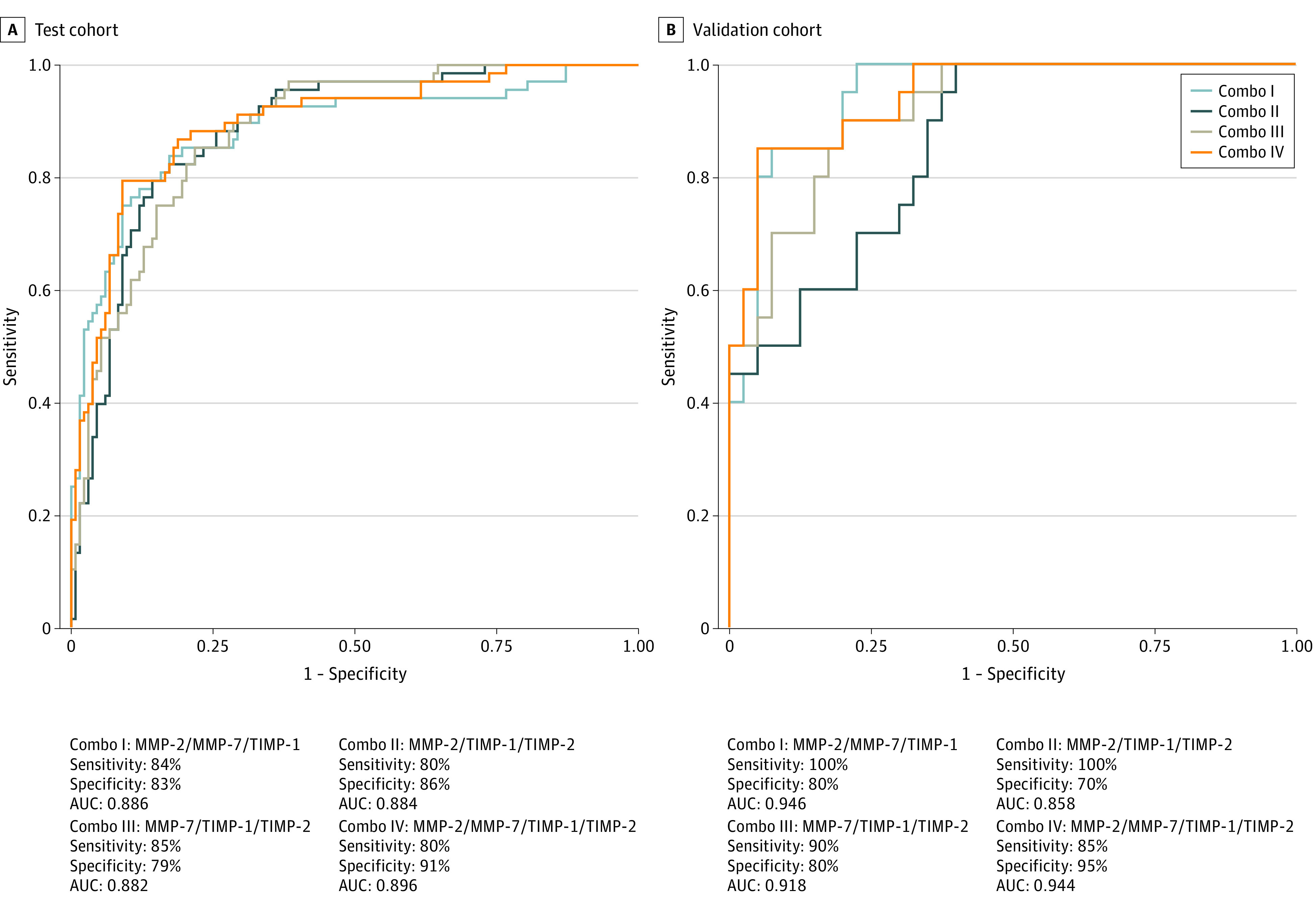
Identification of Biomarkers Showing the Strongest Association Using a Combination of Matrix Metalloproteinases (MMPs) and Tissue Inhibitors of Matrix Metalloproteinases (TIMPs) in Patients With Active Tuberculosis Combination of receiver operating characteristic (ROC) model analysis shows the MMP/TIMP signatures that exhibited the highest accuracy in discriminating cases and controls. ROC curves for comparing multiple markers and their combinations between cases vs controls in the test cohort (A) and validation cohort (B) are shown. AUC indicates area under the curve; Combo, Combi ROC (receiver operating characteristic).

As shown in Figure 3 and eFigure 2 in the Supplement, plasma signatures of MMP-2/MMP-7/TIMP-1 (area under the curve [AUC], 0.886; sensitivity, 84%; and specificity, 83% in the test cohort and AUC, 0.946; sensitivity, 100%; and specificity, 80% in the validation cohort), MMP-2/TIMP-1/TIMP-2, MMP-7/TIMP-1/TIMP-2, and MMP-2/MMP-7/TIMP-1/TIMP-2 exhibited significant discriminatory ability to distinguish cases and controls (eg, for MMP-2/MMP-7/TIMP-1/TIMP-2: AUC, 0.944; sensitivity, 85%; and specificity, 95%). Thus, plasma levels of MMPs and TIMPs may be predictive biomarkers of adverse treatment outcomes in patients with PTB.

## Discussion

Biomarker research is progressing in the areas of differentiating active TB from latent infection, predicting the risk of progression to clinical disease, identifying the response to treatment and relapse, and providing accurate end points for clinical trial of new drugs and vaccines.^26,27,28^ Prognostic TB biomarkers would need to identify individuals with PTB at risk for adverse treatment outcomes, including treatment failure, relapse, and death.^5,7^ Prognostic biomarkers would also help in shortening the duration of chemotherapy in those individuals who are not at risk for these outcomes, because there is evidence that a shorter course of chemotherapy would be sufficient for most individuals with PTB.^29,30^ Another advantage of blood-based biomarkers is the potential for transition to point-of-care tests that could circumvent the need for more expensive and sophisticated instruments.

The aim of the present study was to investigate the capacity of baseline soluble protein biomarkers to predict TB treatment outcomes in 2 different cohorts of Indian adults with TB. The evidence from this study indicates that a variety of plasma MMPs and TIMPs were associated with important differences in cases and controls and may add value to clinical and bacteriologic parameters in identifying individuals at risk for treatment failure, recurrence of TB, or death. Previous studies have mainly relied on clinical and bacteriologic parameters (including baseline time to positivity in culture and month 2 culture status) to predict adverse treatment outcomes.^31,32,33^ However, these parameters do not exhibit high specificity and sensitivity in prediction of outcomes.^34^ We examined a variety of MMPs and TIMPs to delineate the differences in the kinetics of expression in cases vs controls. Our study results showed that 4 different MMPs (MMP-1, MMP-2, MMP-7, and MMP-9) and 2 TIMPs (TIMP-1 and TIMP-2) were associated with increased risk of adverse treatment outcomes in the test and validation cohorts. Our study also showed that the increased risk of adverse outcomes was independent of most clinical, radiologic, and epidemiologic variables. Thus, our study provides novel correlates of risk that could serve as predictors of adverse treatment outcomes at baseline.

In a variety of settings, MMPs and TIMPs are known biomarkers of pulmonary and extrapulmonary TB.^10,11,20^ Several MMPs are upregulated in blood, sputum, and bronchoalveolar lavage of individuals with active TB.^15,35,36,37,38,39^ In addition, MMP levels correlate with lung pathology, with plasma concentrations of several MMPs being associated with radiologic abnormalities.^15,35,36,37,38,39^ Moreover, MMPs are also known biomarkers of disease severity and treatment responses in patients who have both TB and diabetes.^40^ The role of TIMPs as biomarkers of TB disease is not fully explored. To our knowledge, this is the first study that provides evidence of an association of baseline levels of MMPs and TIMPs with adverse treatment outcomes. We extended our study to evaluate plasma signatures with combinations of MMPs and TIMPs to assess optimal biosignatures. Our data revealed that combinations of MMPs and TIMPs have high accuracy in classifying favorable vs adverse treatment outcomes in the 2 cohorts.

Our study provides a promising beginning to unravel the effect of novel non–sputum-based prognostic disease biomarkers in patients with TB. Previous studies on correlates of risk for adverse treatment outcomes have relied on either transcriptional or complex immune or metabolic signatures.^41,42,43,44^ Moreover, correlates of risk studies in plasma/serum have been performed mostly in HIV-infected populations in Africa.^45,46^

### Limitations

Our study has 2 limitations. It was performed with a moderate sample size, and it included all the adverse outcomes under a single umbrella.

## Conclusions

This case-control study is one of the first to be performed in a population without HIV infection in India. Based on the results of this study, further refinement and validation of signatures of TB treatment response are warranted to enable their use to guide individually tailored TB therapy and as readouts in efficacy studies of new treatment regimens and strategies. In summary, our data reveal that MMPs and TIMPs may be prognostic biomarkers of adverse treatment outcomes in patients with PTB.

## Notes

Supplement. **eFigure 1.** Elevated Baseline Plasma Levels of MMPs and TIMPs in Cases in the Test and Validation Cohorts **eFigure 2.** Receiver Operating Characteristic Analysis to Estimate the Discriminatory Power of Systemic MMPs and TIMPs With Bad Outcomes and Control **eTable.** Demographic and Clinical Characteristics of the Study Population—Validation Cohort
