# Supplementary material for: Association of Plasma Matrix Metalloproteinase and Tissue Inhibitors of Matrix Metalloproteinase Levels With Adverse Treatment Outcomes Among Patients With Pulmonary Tuberculosis
Source: JAMA Netw Open. 2020 Dec 1;3(12):e2027754. doi: 10.1001/jamanetworkopen.2020.27754 (PMC7709089; doi:10.1001/jamanetworkopen.2020.27754)
Supplement: Supplement. — eFigure 1. Elevated Baseline Plasma Levels of MMPs and TIMPs in Cases in the Test and Validation Cohorts eFigure 2. Receiver Operating Characteristic Analysis to Estimate the Discriminatory Power of Systemic MMPs and TIMPs With Bad Outcomes and Control eTable. Demographic and Clinical Characteristics of the Study Population—Validation Cohort [file jamanetwopen-e2027754-s001.pdf]

# Supplementary Online Content

Kumar NP, Moideen K, Nancy A, et al. Association of plasma matrix metalloproteinase and tissue inhibitors of matrix metalloproteinase levels with adverse treatment outcomes among patients with pulmonary tuberculosis. *JAMA Netw Open*. 2020;3(12):e2027754. doi:10.1001/jamanetworkopen.2020.27754

**eFigure 1.** Elevated Baseline Plasma Levels of MMPs and TIMPs in Cases in the Test and Validation Cohorts

**eFigure 2.** Receiver Operating Characteristic Analysis to Estimate the Discriminatory Power of Systemic MMPs and TIMPs With Bad Outcomes and Control

**eTable.** Demographic and Cinical Characteristics of the Study Population—Validation Cohort

This supplementary material has been provided by the authors to give readers additional information about their work.

©2020 Kumar NP et al. *JAMA Network Open*.

**eFigure 1. Elevated Baseline Plasma Levels of MMPs and TIMPs in Cases in the Test and Validation Cohorts**

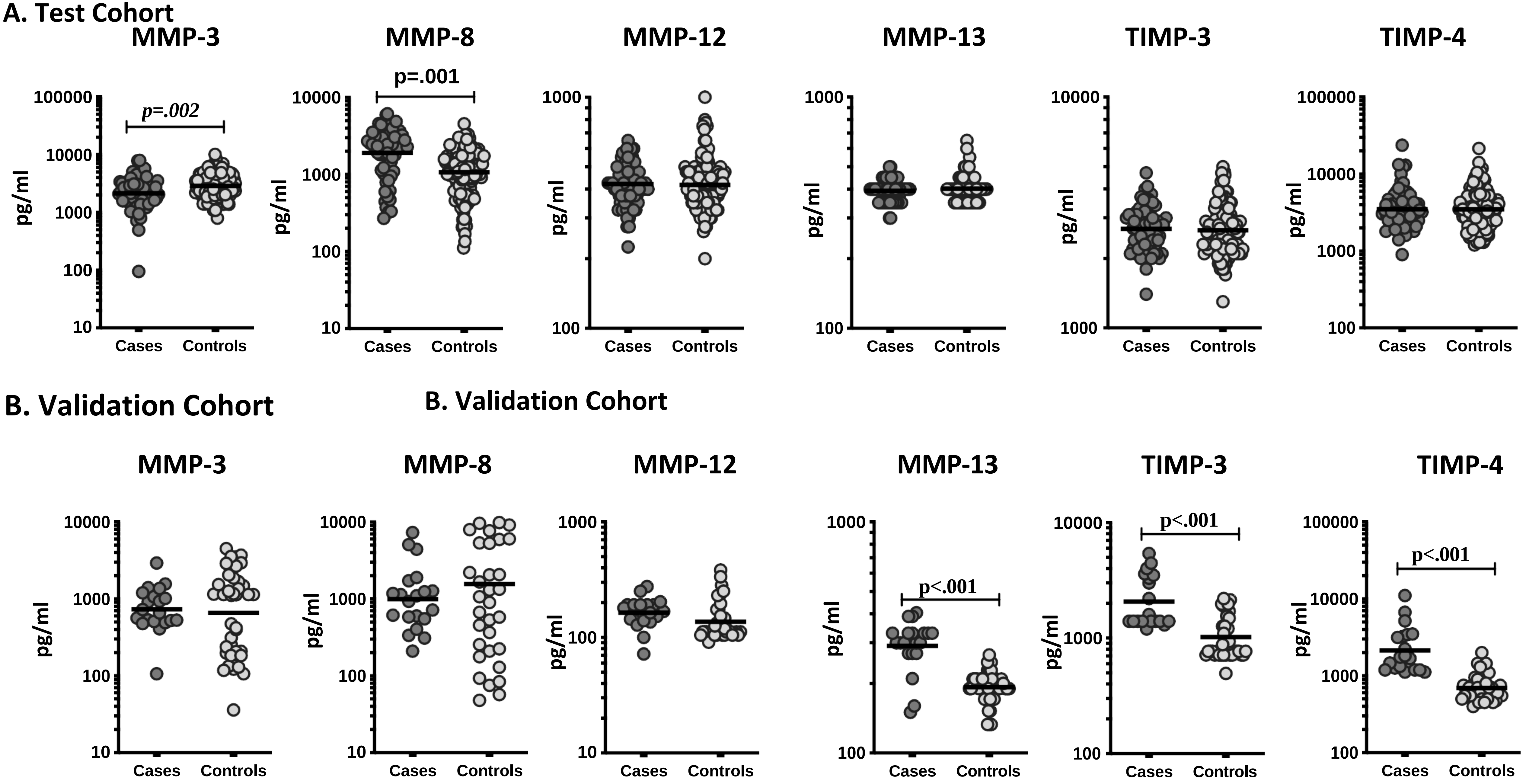


©2020 Kumar NP et al. *JAMA Network Open*.

eFigure 2. Receiver Operating Characteristic Analysis to Estimate the Discriminatory Power of Systemic MMPs and TIMPs With Bad Outcomes and Control

## A. Relapse Cases vs Controls

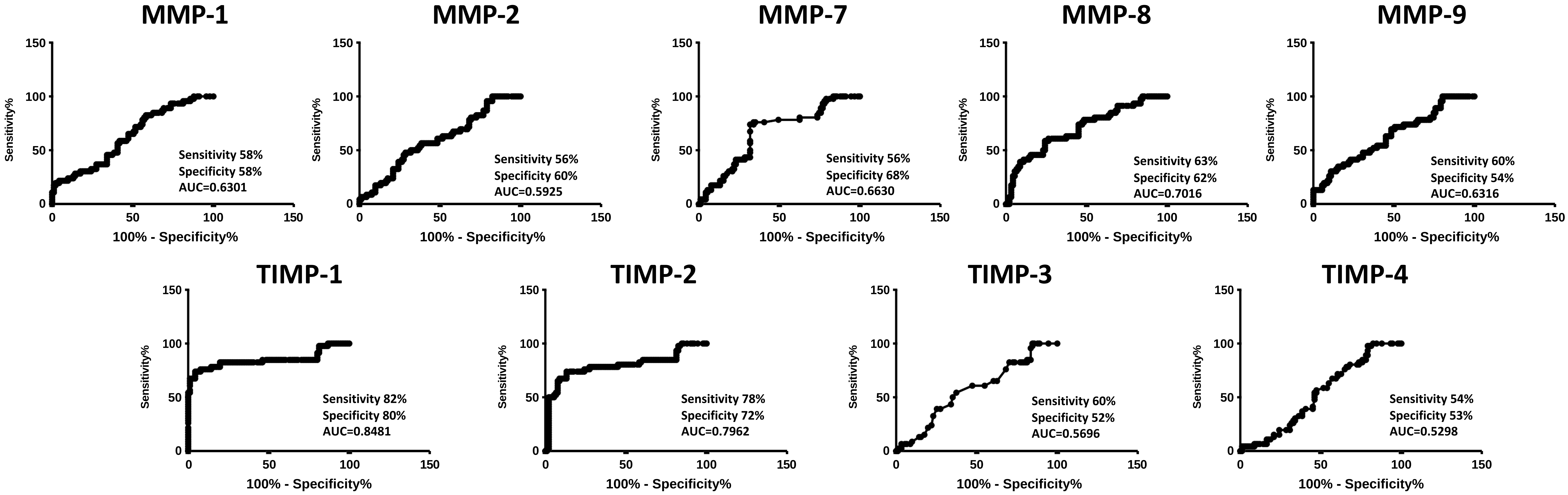


## B. Treatment Failure Cases vs Controls

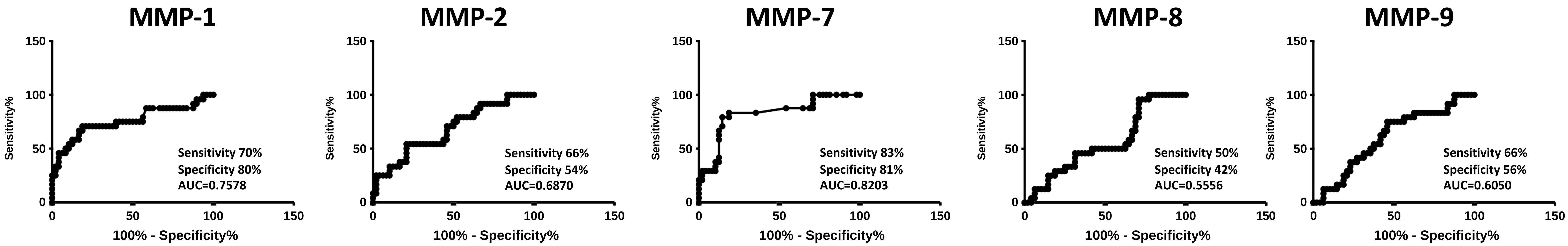


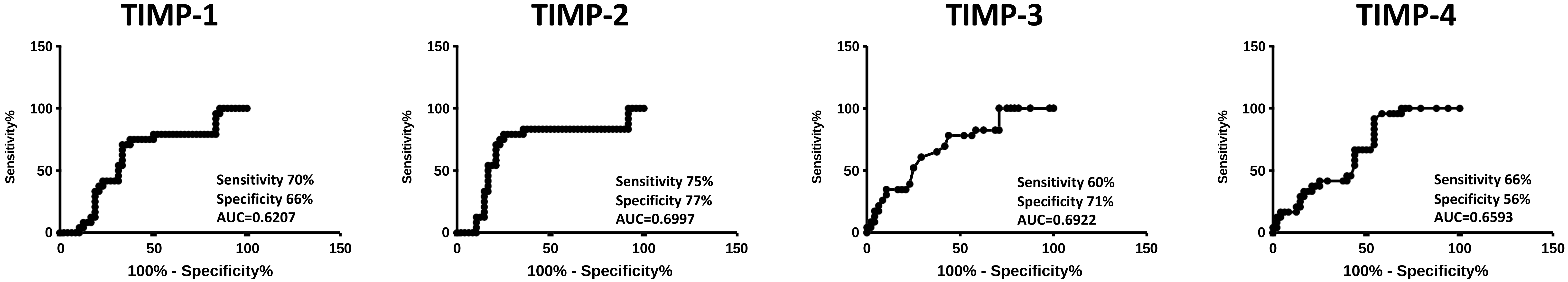


©2020 Kumar NP et al.
*JAMA Network Open.*

**eTable. Demographic and clinical characteristics of the study population – Validation cohort**

| Characteristic | All (n=60) | Cases (n=20 [34%]) | Controls (n=40 [66%]) | P Value |
|---|---|---|---|---|
| Age, y, median | 45 (19 – 61) | 45 (20 - 48) | 40 (19 - 61) | 0.1297 |
| **Gender** | | | | |
| Male | 51 [85%] | 19 [95%] | 32 [80%] | |
| Female | 9 [15%] | 1 [5%] | 8 [20%] | 0.1250 |
| **Cavity** | | | | |
| Yes | 27 [45%] | 10 [50%] | 17 [43%] | |
| No | 33 [55%] | 10 [50%] | 23 [57%] | 0.5820 |
| **Smear Grade** | | | | |
| 1+ | 22 [36%] | 7 [35%] | 15 [38%] | |
| 2+ | 21 [35%] | 7 [35%] | 14 [35%] | |
| 3+ | 17 [29%] | 6 [30%] | 11 [27%] | 0.9743 |
| **Culture Grade** | | | | |
| 1+ | 20 [33%] | 7 [35%] | 13 [33%] | |
| 2+ | 20 [33%] | 7 [35%] | 13 [33%] | |
| 3+ | 20 [33%] | 6 [30%] | 14 [34%] | 0.9277 |

©2020 Kumar NP et al. *JAMA Network Open*.
